# Breaking Bread: the Functions of Social Eating

**DOI:** 10.1007/s40750-017-0061-4

**Published:** 2017-03-11

**Authors:** R. I. M. Dunbar

**Affiliations:** Department of Experimental Psychology, University of Oxford, South Parks Road, Oxford, OX1 3UD UK

**Keywords:** Support clique, Happiness, Trust, Social engagement, Social bonding

## Abstract

Communal eating, whether in feasts or everyday meals with family or friends, is a human universal, yet it has attracted surprisingly little evolutionary attention. I use data from a UK national stratified survey to test the hypothesis that eating with others provides both social and individual benefits. I show that those who eat socially more often feel happier and are more satisfied with life, are more trusting of others, are more engaged with their local communities, and have more friends they can depend on for support. Evening meals that result in respondents feeling closer to those with whom they eat involve more people, more laughter and reminiscing, as well as alcohol. A path analysis suggests that the causal direction runs from eating together to bondedness rather than the other way around. I suggest that social eating may have evolved as a mechanism for facilitating social bonding.

## Introduction

Feasting, the gathering together in groups for a communal meal, has long been of interest to both archaeologists (Hayden 1996, 2014; Whitehead 2000; Bray 2003; Jones 2007) and anthropologists (Rappoport 1968; Strathern 1971; Young 1971). Much of the focus of this interest has been on competitive feasting as a form of display, in which debts are created, status advertised, rituals celebrated and, in some cases, excess food disposed of. Feasting on this scale may date back only as far as the Neolithic and the agricultural production of food surpluses. However, foragers also feast, even though the scale is usually very different and commonly involves the consumption of certain kinds of food that come in large packets with a limited shelf life (Harris 1971; Lee 1972).

While special occasions of this kind inevitably attract attention, in fact feasting on this scale represents the tip of an iceberg of communal eating that mainly focusses on family and friends. Family meals are widespread and commonplace in all cultures, and inviting friends or visitors to dine remains a regular social activity in most societies – with communal eating with guests being widely regarded as both the height of hospitality and an important way of getting to know people. Even in times when fast food dominates everyday culture, sitting down to eat with family and friends continues to be seen as important and desirable.

Why do we do this? There is no intrinsic reason (other than the convenience of bulk cooking) that makes communal eating essential, especially for hunter-gatherers. Given that social meals inevitably take longer than eating alone, what is it about communal eating that is so beneficial? Potential benefits can be identified at three different levels: communal, networking and personal. These can be identified, respectively, with (a) building wider community and inter-community relationships, usually on a large scale but at infrequent intervals (‘feasting’ in the more conventional sense), (b) making and reinforcing (i.e. servicing) friendship and family relationships, usually on a modest scale and at more frequent (perhaps even daily) intervals and (c) at the personal level in terms of health benefits. The first two relate to indirect fitness benefits that accrue through the formation of mutual alliances at different levels (between versus within community), while the third relates to direct fitness in terms of health benefits that arise from well-formed social relationships.

Friendships provide important health benefits, although the significance of these has only recently been appreciated. There is now considerable evidence, for example, to suggest that the size and quality of one’s social network has very significant consequences for one’s health, susceptibility to illness (and even death), wellbeing and happiness (Holtzman et al. 2004; Min et al. 2007; Rodriguez-Laso et al. 2007; Fowler and Christakis 2008; Dominguez and Arford 2010; Pinquart and Duberstein 2010; Holt-Lunstad et al. 2010; Liu and Newschaffer 2011; Chou et al. 2012; Tilvis et al. 2012; Oesch and Dunbar 2015). We also know that activities such as laughter, singing and dancing all lead to an enhanced sense of bonding towards those with whom one does these activities (Dunbar et al. 2012; Pearce et al. 2015; Tarr et al. 2015, 2016; Manninen et al., submitted), mainly because they trigger the endorphin system in the brain that underpins primate social bonding (Panksepp et al. 1997; Curley and Keverne 2005; Dunbar 2010; Machin and Dunbar 2011). Since endorphins are involved in the control of feeding (Bakshi and Kelley 1993; Zhang and Kelley 2000; DiFeliceantonio et al. 2012), the very fact of eating might itself trigger the endorphin system and promote bonding, and doing so socially may lead to the same kind of enhanced endorphin effects from behavioural synchrony that have been noted in physical exercise (Cohen et al. 2010). Hence, people who eat often with others might be expected to have larger social networks and be happier and more satisfied with their lives, as well as being more engaged with their communities.

My focus will be on the more modest everyday social scale rather than on large scale communal feasts as such, not least because communal feasts happen only irregularly and hence are difficult to study with sufficient frequency to provide meaningful samples. Social eating, on the other hand, is a daily activity across all cultures, with regular midday or evening social meals being a near-universal practice. I use data from a national stratified survey carried out in the UK to ask two principal questions. First, are respondents who eat regularly with others more likely to feel happier, more satisfied with life and more engaged with their communities, and have a larger number of friends and family on whom they can depend for support than respondents who more often eat alone? Second, does having a recent evening meal with someone other than a household member result in an increased feeling of closeness to that person, and does the strength of this effect depend on what behaviours had occurred during the meal?

## Methods

As part of a collaboration with Big Lunch project ( http://www.thebiglunch.com), a national stratified UK sample was commissioned through the polling agency OnePoll. A panel sample of 2000 adults aged over 18 years, balanced for regional distribution, age and gender, was sampled in one week in April 2016. In addition to demographic information (age, gender, nearest city), respondents were asked to rate how many meals they ate alone during the week and (on 7-point Likert scales) how often they had eaten meals with different members of their extended network; they were also asked to rate, on 10-point analogue scales, how satisfied they felt with life, how worthwhile they felt their life to be, how happy they had been the day before, and (using the 7-point Inclusion-of-Other-in-Self, IOS, scale: Aron et al. 1992) how engaged they felt themselves to be with their local community. Finally, they were asked how many close friends and family they had whom they felt they could depend on for emotional, social and financial support if they needed it (with six options to choose from: 0, 1, 2–3, 4–5, 6–10, 11+). These values were chosen on the basis of previous studies which indicate that this layer of the social network has a very consistent average of five individuals (see Dunbar and Spoors 1995; Hill and Dunbar 2003; Sutcliffe et al. 2012; Burton-Chellew and Dunbar 2015).

To determine what it is about eating together that contributes to the sense of engagement we have when we eat socially, respondents were asked to recall the last time they had had a midday and an evening meal with someone they didn’t live with and to rate (on a scale 0 = not at all to 10 = a great deal) how much closer they felt to the people concerned afterwards. In respect of the evening meal, they were also asked to say how many people were present (2, 3, 4 or 5+, including themselves) and, on a simple binary choice, whether or not any of the following had occurred during the meal: laughter, reminiscences, jokes, singing, dancing, party games, drinking alcohol or eating chocolate. Laughter, singing, dancing and storytellling are all known to trigger the endorphin system (Dunbar et al. 2012; Pearce et al. 2015; Tarr et al. 2016; Dunbar et al. 2016), the main pharmacological factor underpinning social bonding in primates and humans (Curley and Keverne 2005; Depue and Morrone-Strupinsky 2005; Machin and Dunbar 2011). Alcohol is also a major trigger of the endorphin system (Naber et al. 1981; Van Ree 1996; Hertz 1997; Gianoulakis 2004) – so much so that an endorphin antagonist such as naltrexone is now the treatment of choice for alcohol addiction (O'Brien et al. 1996; Saland et al. 2008). Chocolate was included to provide a non-endorphin control, and hence to stand simply for eating: all the other activities (and especially alcohol) trigger the release of endorphins.

## Results

**Fig. 1:**
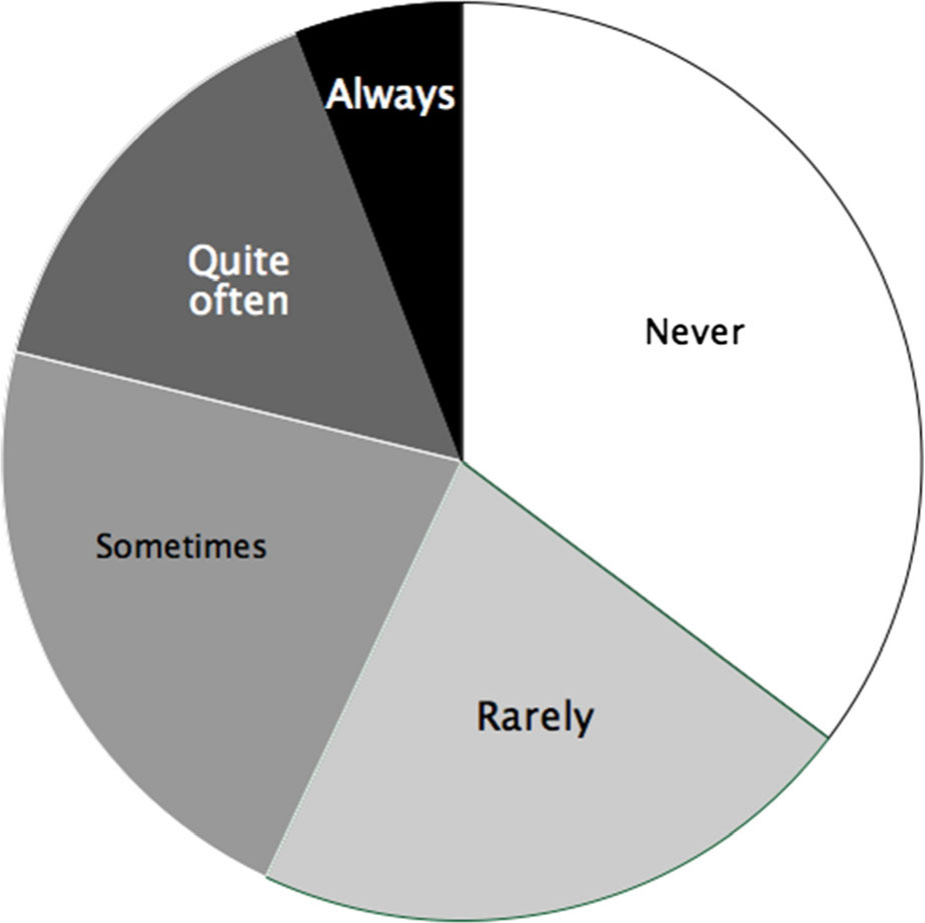
Frequency with which respondents reported having evening meals alone

**Fig. 2:**
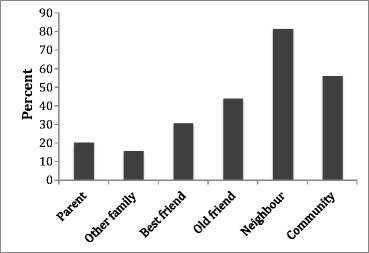
Percentage of respondents who had not had a meal in the previous 6 months with different categories of people they know

As one might expect, most people (93%) did have meals with family and friends at least sometimes (Fig. 1). Even so 15% said they hadn’t had a meal with another family member in the last six months, 30% said they hadn’t done so with a best friend in the last six months, and 45% hadn’t done so with an old friend (Fig. 2). Women were more likely to have eaten with all these people than men, although in each case the difference was modest and not significant. Respondents were also asked how often they ate with people other than their immediate family. Nearly 70% said they had never had a meal with a neighbour, 15% had never had a meal with a work colleague, 32% had never done so with a boss or manager, and 37% had never had a meal with a community group. As many as 65% felt there was someone they should make more effort to see or spend time with, and 75% thought this was best done by sharing a meal.

**Fig. 3:**
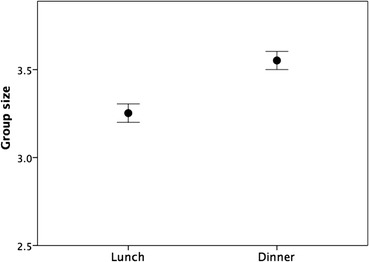
Mean (±2SE) number of people at the respondents’ last lunch or dinner

On average, respondents last had lunch with a good friend or family member only within the last month. The same was true of the last time they had had an evening meal with a close friend or family member. Lunch groups were significantly smaller than dinner groups (within-individual matched pairs t-test, t_1914_ = 10.14, *p* < 0.0001), and were skewed towards pairs (including the respondent). Only 21% of lunch groups contained more than four individuals, and only 27% of dinner groups did so. Nonetheless, the average size of lunch and dinner groups (Fig. 3: means of 3.3 ± 1.2 and 3.6 ± 1.3 respectively, including the respondent) is virtually identical to the average size of free-forming conversational groups (~3.5: Dunbar et al. 1995; Dunbar 2016; Krems et al. 2016; Dahmardeh and Dunbar 2017).

In terms of life satisfaction, 69% felt satisfied with their life, 67% had been happy on the day before and 70% felt that what they did in their life was worthwhile. However, only 46% of people trusted the people they met, and only 30% felt engaged with their local community. These indices of life satisfaction all correlated highly with each other (Kendall’s 0.756 ≥ τ ≥ 0.106, *p* < 0.01). The two sexes did not differ on any of these indices (F_1,1998_ ≤ 0.95, *p* ≥ 0.331).

**Fig. 4:**
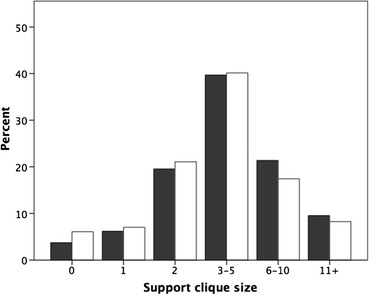
Distribution of size of support clique for men (*grey bars*) and women (*white bars*)

**Fig. 5:**
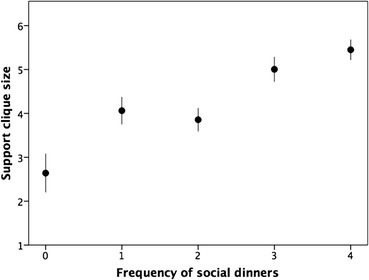
Mean (±95% CI) number of close friends and family that a respondent could rely on plotted against how often they reported eating their evening meal with other people. X-axis: 0 = never; 1 = rarely; 2 = sometimes; 3 = quite often; 4 = very often

On average, respondents said they had 4.63 ± 3.06 people they could count on for emotional and other forms of support and help (Fig. 4). This value is very close to the typical value of ~5 individuals (usually split evenly between family and friends) reported for the support clique in many previous studies (Dunbar and Spoors 1995; Hill and Dunbar 2003; Stiller and Dunbar 2007; Sutcliffe et al. 2012; Burton-Chellew and Dunbar 2015). As found in most previous studies, females had significantly more close friends than males (F_1,1998_ = 6.63, *p* = 0.01), although in practice the difference (on average, ~0.5 individual) is small. Figure 5 plots the size of the support clique against how often respondents ate their evening meal with someone else. Those who rarely ate socially had many fewer friends and family they could count on for moral, social, emotional or financial support when they needed it. Typically, across the population as a whole this averages about five individuals, but those who mostly ate alone had as few as half this number.

**Fig. 6:**
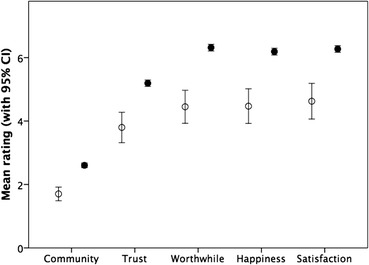
Mean (95% CI) rating of being engaged with the local community, level of trust in local community, worthwhileness of life, happiness on day before and satisfaction with life, for respondents who always ate their evening meals alone (open symbols) or who at least sometimes ate with others (filled symbols). Ratings are on a 1–10 scale, except Community (1–7)

Figure 6 plots the mean rating for the five social indices as a function of whether respondents normally ate their evening meal alone or did so socially at least sometimes. Note that while the four indices on the right are rated on a 1–10 scale, being engaged with one’s local community (Community) is rated on a 1–7 scale using the IOS. In all cases, those who ate socially at least sometimes gave significantly higher ratings than those who always ate alone (F ≥ 37.4, df = 1,1712–1870 depending on index, *p* < 0.001).

A multiple regression with the frequency of social evening meals as the dependent variable and these five social indices plus support clique size as independent variables yields a significant model (F_6,1991_ = 43.993, *p* < 0.0001), with life satisfaction, engagement with the community and support clique size as the only significant factors (Table 1). Note that engagement with the local community differs from the other variables in that it is negatively (albeit only weakly) correlated with the frequency of social meals; a factor analysis suggests that this index loads on a separate factor to the four other indices. Including sex and age in the model does not change the results, and sex itself was not a significant factor. (For all the indices except Community engagement, there is a significant, but very weak, linear relationship with age; adding a quadratic term for age does not improve the fit.) A backwards stepwise regression yields significant results for all possible models with marginal differences in fit; the variables most likely to be excluded are happiness and support clique size, suggesting that these are not especially reliable predictors of the frequency of eating socially.

**Table 1:** Multiple regression model of the predictors of the frequency with which respondents had evening meals with other people

Variable	β*	t^§^	p
Life satisfaction	0.129	2.80	0.005
Happy yesterday	0.058	1.35	0.176
Life worthwhile	0.052	1.34	0.182
Trust people	0.039	1.64	0.100
Engaged with community	-0.044	-2.00	0.046
Support clique size	0.178	7.78	<0.001

* standardised slope; § df = 1991; Model: F_6,1991_ = 43.993, *p* < 0.001

**Fig. 7:**
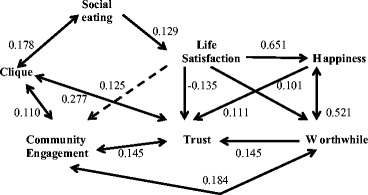
Path analysis of the relationship between the main variables in Table 1. All significant partial standardized coefficients are shown. *Solid lines*: positive coefficients; *dashed lines*: negative coefficients. Numbers beside the lines are the standardized coefficients. *Single headed arrows* indicate cases where one coefficient was significant and the reciprocal coefficient not, or where one was at least double the other. *Double arrows* indicate cases where both coefficients were significant and of similar magnitude; in these cases, the larger of the two coefficients is given

To provide some insight into causality, I ran a path analysis using the partial standardized slope coefficients from the multiple regression analyses for each of the variables in Table 1 (excluding age and sex, which are not of particular interest and, in any case, were not significant). All significant partial coefficients are shown in Fig. 7. Cases where there was a clear directional difference (one coefficient was significant and its reciprocal not, or one coefficient was at least twice the value of the other) are indicated by a single headed arrow indicating causal direction; negative relationships are indicated by dashed arrows. While the overall pattern is complex and involves many feedback loops and multivariate effects, the path analysis suggests a clear causal pathway in which eating social dinners both correlates with clique size and increases life satisfaction, and that enhanced satisfaction in turn increases one’s happiness, trust in others and sense that life is worthwhile, while at the same time negatively influencing engagement in the community. Notice how clique size, satisfaction, happiness and a worthwhile life all increase trust in others. Finally, number of close friends, trustingness and the feeling that life is worthwhile all positively enhance engagement in the community (independently of life satisfaction). It is noteworthy that the relationship between clique size and eating together is bi-directional. This perhaps suggests that one eats with people as a way of creating and servicing relationships, and in consequence those who have many friends are likely to eat socially more often.

Asked if sharing a meal was a good way to bring people closer together, 76.4% said it was (with 18.1% not sure). When meeting someone new, women exhibited a greater preference for a lunchtime meeting (41% against 20% for an evening event), whereas men had a slight preference for the evening (25% vs 30% respectively). In contrast, 34% of people felt that an evening meal would be the better occasion to meet up with an old friend or family member, with 30% opting for a lunch event. Although women still preferred a lunchtime event (34%), a much higher proportion of them (32%) opted for an evening event in this case. Only 4% of people said they wouldn’t suggest a meal as a way to meet. A matched pairs t-test suggests that respondents felt significantly closer to a fellow diner after an evening meal than they did after a midday meal (t_1732_ = −6.058, *p* < 0.0001).

**Fig. 8:**
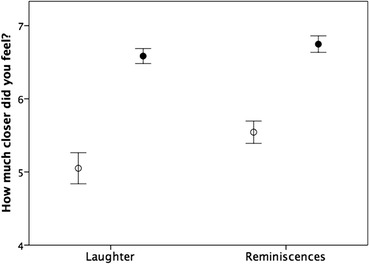
Mean (±2 SE) rating of how much closer respondents felt to the other people with whom they had an evening meal depending on whether (filled symbols) or not (unfilled symbols) the meal was accompanied by laughter or reminiscences

It remains unclear whether it is eating together per se or something else that happens during the meal that creates the sense of social engagement. A definitive answer to this question would need an experimental design that was not possible in a survey study. Nonetheless, we can gain some insights by considering some of the activities that occur during social meals. Respondents were asked to rate how much closer they felt to the people with whom they had had their most recent evening meal (on a 0 = not at all to 10 = a great deal), how many people had been present (four categories) and (as binary presence/absence variables) whether laughter, reminiscences, jokes, singing, dancing, party games, alcohol drunk or chocolate eaten had occurred. The frequencies with which each of these occurred are indicated in Table 2. An analysis of variance, with sense of closeness as the dependent variable and all nine variables as factors, was highly significant (F_11,17_ = 25.89, *p* < 0.0001). However, only four of the variables had significant independent effects: number of diners, laughter, reminiscences, and the consumption of alcohol (Table 2). Of these, laughter and reminiscences had by far the strongest effects (Fig. 8). Including sex and age in the model does not change the results (though in this case there was a significant effect due to sex, with females responding more strongly: *p* = 0.045). Notice that the frequency of jokes was not of itself important; rather, even though jokes might be used because they trigger laughter, it seems to be the laughter itself, whatever it is triggered by, that creates the sense of bonding. Similarly, the consumption of chocolate (and hence, by implication, what was eaten) had no detectable effect.

**Table 2:** ANOVA of factors influencing increased sense of feeling closer to dinner companion after eating with them in the evening

Variable	Frequency (%)^¶^	Mean square	F*^§^	p
Number of diners	(mean = 3.6^#^)	21.5	6.8	<0.001
Laughter	67.9	188.8	59.5	<0.001
Jokes	22.3	7.8	1.8	0.174
Reminiscences	51.5	120.0	37.8	<0.001
Party games	4.2	0.5	0.2	0.679
Singing	4.1	7.8	2.5	0.118
Dancing	5.1	0.5	0.2	0.698
Alcohol	53.9	37.1	11.7	0.001
Chocolates	12.8	0.1	<0.1	0.895

^¶^Percent of dinners at which behaviour indicated occurred. # including respondent

* df = 1,1718, except number of diners where df = 3,1718; § full model: F_11,1718_ = 25.9, *p* < 0.0001

## Discussion

These survey data allow us to conclude (1) that people who eat socially are more likely to feel better about themselves and to have a wider social network capable of providing social and emotional support, (2) that eating with someone in the evening makes one feel closer to them than eating with them at midday and (3) that evening meals at which laughter and reminiscences occur and alcohol is drunk are especially likely to enhance feelings of closeness. Neither age nor sex had especially significant or noteworthy effects in any of these respects, other than to confirm the fact that, as noted in many previous studies, women typically have more close friends than men do. Intriguingly, the analyses suggest that feeling engaged with the local community is dimensionally orthogonal (i.e. unrelated) to feelings of personal happiness and satisfaction with one’s circumstances.

Causality is always difficult to determine from correlational data. One possibility is obviously that people who eat regularly with others have a wider circle of close friends as a result and thus feel more embedded in their communities. However, we cannot exclude the alternative possibility that having a wider circle of friends or being embedded in the community for other reasons (such as being an extravert) causes individuals to eat socially more often. Nonetheless, in their more qualitative responses, a significant proportion of respondents typically felt that having a meal together was an important way of making or reinforcing friendships, suggesting that this may be why they ate with others. Indeed, the fact that having eaten together increased respondents’ sense of closeness to the person concerned (Table 2) would be difficult to interpret as reversed causality: it is logically impossible for feeling closer after eating to cause one to eat with that person beforehand since, conventionally, causes have to come before, rather than after, their effects. One may have a meal with someone *in the hope* that it will increase one’s feeling of closeness, but whether or not one actually feels closer afterwards is likely to depend on the meal itself and not on the expectation. In fact, the most convincing evidence for causal direction is provided by the path analysis of Fig. 2, which clearly favours the claim that eating with someone generates not only more bonded relationships but also enhances one’s sense of contentedness and embedding within the community. Ultimately, however, experimental studies will be needed to confirm causality.

Exactly what it is about eating together that causes these effects isn’t completely clear, though the analysis in Table 2 suggests that laughter and the telling of tales (in this case, reminiscences) play an important role, aided and abetted by the consumption of alcohol (see also Dunbar et al. 2017). Both laughter (Manninen et al.,  submitted) and alcohol (Gianoulakis and Barcomb 1987; Gianoulakis 2004) are known to trigger the endorphin system, the principal psychopharmacological mechanism that underpins primate and human social bonding (Dunbar 2010; Machin and Dunbar 2011). Although the potential role of eating was not directly tested here other than in respect of chocolate consumption, endorphin activation is known to be associated with feeding behavior (Bakshi and Kelley 1993; Zhang and Kelley 2000; DiFeliceantonio et al. 2012). It is also possible that the endorphin system is triggered by certain ingredients (e.g. chili or other spices) that stress the digestive system. If so, it is possible that endorphins could be part of the mix even when people simply eat together without engaging in any of the activities in Table 2. However, it may just be that the effect derives simply from the opportunity that food provides for engaging in the activities (laughter, reminiscences, etc) that do trigger the endorphin system.

One potentially important finding is that evening meals are regarded as significantly more appropriate or valuable for building friendships than eating at midday. Casual observation suggests that many of our most important social activities happen in the evening; doing these things at night seems to have an added ‘magic’. Yet almost no one seems to have commented on this. In a recent analysis of conversational topics among San hunter-gatherers, Wiessner (2014) noted that social topics predominate in evening conversations, whereas daytime conversations are typically more factual and functional. Dunbar (2014a, b) drew attention to the fact that shifting social time into the evening when firelight could be used to extend the waking day was likely to have been a crucial means whereby humans mitigated the time budget constraints that would otherwise have prevented further increases in brain size and social community size. By moving all one’s social time to the evening when one can eat and talk around the campfire would have freed off a significant amount of daytime for foraging and other essential activities. The semi-dark may have had made evening social activities more ‘magical’ and engaging, and given rise to a preference for carrying out such activities in the evening. It would also, of course, have placed a premium on vocal channels of communication: gestural communication is less effective in the dark (Dunbar 2014b, 2017).

Over the past decade or so, considerable evidence has emerged that the number and quality of close friendships has a significant and direct impact on health, wellbeing and even survival (see, among a great many others, Waxler-Morrison et al. 1991; Flinn and England 1995; Sayal et al. 2002; Kikusui et al. 2006; Kana’iaupuni et al. 2005; Charuvastra and Cloitre 2008; Holt-Lunstad et al. 2010; Pinquart and Duberstein 2010; Liu and Newschaffer 2011; Chou et al. 2012; Tilvis et al. 2012). Indeed, a similar effect has been noted in several baboon populations: the size and quality of a female’s social network (especially that with other adult females) correlates with her fertility and offspring survival rates (Silk et al. 2003, 2009, 2010) and with her ability to cope with stressful events (Wittig et al. 2008). Thus, not only may social dining have implications for how many friends one has, but this is in turn likely to have significant consequences at the level of individual health and welfare, adding further significant fitness benefits. It is not clear how this effect is produced. One possibility may be that family and friends provide support and assistance in time of need (Spence 1954; McCullogh and York Barton 1991; Grayson 1993), and hence allow one to feel more relaxed in stressful situations. However, there is also evidence that endorphins ‘tune’ the immune system (Sarkar et al. 2012). If so, it is possible that eating together may have health and survival benefits both directly and, through bigger and better social networks, indirectly.

## References

[CR1] Aron A, Aron EN, Smollan D (1992). Inclusion of other in the self scale and the structure of interpersonal closeness. Journal of Personality and Social Psychology.

[CR2] Bakshi VP, Kelley AE (1993). Feeding induced by opioid stimulation of the ventral striatum: role of opiate receptor subtypes. Journal of Pharmacology and Experimental Therapeutics.

[CR3] Bray TL (2003). The archaeology of food and feasting in early states and empires.

[CR4] Burton-Chellew M, Dunbar RIM (2015). Romance and reproduction are socially costly. Evolutionary Behavioral Science.

[CR5] Charuvastra A, Cloitre M (2008). Social bonds and posttraumatic stress disorder. Annual Review of Psychology.

[CR6] Chou A, Stewart S, Wild R, Bloom J (2012). Social support and survival in young women with breast carcinoma. Psycho-Oncology.

[CR7] Cohen E, Ejsmond-Frey R, Knight N, Dunbar RIM (2010). Rowers’ high: behavioural synchrony is correlated with elevated pain thresholds. Biology Letters.

[CR8] Curley JP, Keverne EB (2005). Genes, brains and mammalian social bonds. Trends in Ecology & Evolution.

[CR9] Dahmardeh M, Dunbar RIM (2017). What shall we talk about in Farsi? Content of everyday conversations in.

[CR10] Depue RA, Morrone-Strupinsky JV (2005). A neurobehavioral model of affiliative bonding: implications for conceptualising a human trait of affiliation. Behavioral and Brain Sciences.

[CR11] DiFeliceantonio AG, Mabrouk OS, Kennedy RT, Berridge KC (2012). Enkephalin surges in dorsal neostriatum as a signal to eat. Current Biology.

[CR12] Dominguez S, Arford T (2010). It is all about who you know: social capital and health in low-income communities. Health Sociology Review.

[CR13] Dunbar RIM (2010). The social role of touch in humans and primates: behavioural function and neurobiological mechanisms. Neuroscience and Biobehavioral Reviews.

[CR14] Dunbar RIM (2014). How conversations round campfires came to be. Proceedings of the National Academy of Sciences USA.

[CR15] Dunbar, R. I. M. (2014b). *Human Evolution*. Harmondsworth: Pelican Books and New York: Oxford University Press.

[CR16] Dunbar RIM (2016). Sexual segregation in human conversations. Behaviour.

[CR17] Dunbar, R.I.M. (2017). Group size, vocal grooming and the origins of language. *Psychonomic Bulletin and Review* (in press). doi:10.3758/s13423-016-1122-6. 10.3758/s13423-016-1122-6 27460463

[CR18] Dunbar RIM, Spoors M (1995). Social networks, support cliques and kinship. Human Nature.

[CR19] Dunbar RIM, Duncan N, Nettle D (1995). Size and structure of freely forming conversational groups. Human Nature.

[CR20] Dunbar RIM, Baron R, Frangou A, Pearce E, van Leeuwen EJC, Stow J, Partridge P, MacDonald I, Barra V, van Vugt M (2012). Social laughter is correlated with an elevated pain threshold. Proceedings of the Royal Society of London.

[CR21] Dunbar RIM, Teasdale B, Thompson J, Budelmann F, Duncan S, van Emde Boas E, Maguire L (2016). Emotional arousal when watching drama increases pain threshold and social bonding. Royal Society Open Science.

[CR22] Dunbar, R.I.M., Launay, J., Wlodarski, R., Robertson, C., Pearce, E., Carney, J. & MacCarron, P. (2017). Functional benefits of (modest) alcohol consumption. *Adaptive Human Behavior and Physiology* (in press). doi:10.1007/s40750-016-0058-4. 10.1007/s40750-016-0058-4 PMC7010365 32104646

[CR23] Flinn M, England B (1995). Childhood stress and family environment. Current Anthropology.

[CR24] Fowler JH, Christakis NA (2008). The dynamic spread of happiness in a large social network. British Medical Journal.

[CR25] Gianoulakis C (2004). Endogenous opioids and addiction to alcohol and other drugs of abuse. Current Topics in Medicinal Chemistry.

[CR26] Gianoulakis C, Barcomb A (1987). Effect of acute ethanol *in vivo* and *in vitro* on the β-endorphin system in the rat. Life Sciences.

[CR27] Grayson DK (1993). Differential mortality and the Donner party disaster. Evolutionary Anthropology.

[CR28] Harris M (1971). Culture, man and nature.

[CR29] Hayden B, Wilson P, Weissner P, Schiefenhövel W (1996). Feasting in prehistoric and traditional societies. Food and the status quest: An interdisciplinary perspective.

[CR30] Hayden B (2014). The power of feasts: From prehistory to the present.

[CR31] Hertz A (1997). Endogenous opioid systems and alcohol addiction. Psychopharmacology.

[CR32] Hill RA, Dunbar RIM (2003). Social network size in humans. Human Nature.

[CR33] Holt-Lunstad J, Smith TB, Bradley Layton J (2010). Social relationships and mortality risk: a meta-analytic review. PLoS Medicine.

[CR34] Holtzman, R.E., Rebok, G. W., Saczynski, J. S., Kouzis, A. C., Wilcox Doyle, K. & Eaton, W. W. (2004). Social network characteristics and cognition in middle aged and older adults. Journal of Gerontology 59B: P278-P284. 10.1093/geronb/59.6.p278 15576855

[CR35] Jones, M. (2007). *Feast: Why humans share food*. Oxford: Oxford University Press.

[CR36] Kana’iaupuni S, Donato K, Thompson-Colon T, Stainbeck M (2005). Counting on kin: social networks, social support, and child health status. Social Forces.

[CR37] Kikusui T, Winslo J, Mori Y (2006). Social buffering: relief from stress and anxiety. Philosophical Transactions of the Royal Society of London.

[CR38] Krems J, Neuberg S, Dunbar RIM (2016). Something to talk about: Are conversation sizes constrained by mental modeling abilities?. Evolution and Human Behavior.

[CR39] Lee RB, Ucko P, Tringham R, Dimbleby GW (1972). Work effort, group structure and land use in contemporary hunter gatherers. Man, settlement and urbanism.

[CR40] Liu L, Newschaffer CJ (2011). Impact of social connections on risk of heart disease, cancer and all-cause mortality among elderly Americans: findings from the second longitudinal study of aging (LSOA II). Archives of Gerontology and Geriatrics.

[CR41] Machin A, Dunbar RIM (2011). The brain opioid theory of social attachment: a review of the evidence. Behaviour.

[CR42] McCullogh JM, York Barton E (1991). Relatedeness and mortality risk during a crisis year: Plymouth colony, 1620-1621. Ethology and Sociobiology.

[CR43] Min S-Y, Whitecraft E, Rothbard AB, Salzer MS (2007). Peer support for persons with co-occurring disorders and community tenure: A survival analysis. Psychiatric Rehabilitation Journal.

[CR44] Naber D, Soble MG, Pickar D (1981). Ethanol increases opioid activity in plasma of normal volunteers. Pharmacopsychiatry.

[CR73] Manninen, S., Tuominen, L., Dunbar, R.I.M., Karjalainen,T., Hirvonen, J., Arponen, E., Hari, R., Jääskeläinen, I.P., Sams, M. and Nummenmaa, L. (submitted). Social laughter triggers endogenous opioid release in humans. 10.1523/JNEUROSCI.0688-16.2017 PMC6596504 28536272

[CR46] O'Brien CP, Volpicelli LA, Volpicell JR (1996). Naltrexone in the treatment of alcoholism: a clinical review. Alcohol.

[CR47] Oesch N, Dunbar R (2015). Influence of kin network on maternal and infant health and illness. Journal of Pregnancy and Child Health.

[CR48] Panksepp J, Nelson E, Bekkedal M (1997). Brain systems for the mediation of social separation-distress and social-reward: evolutionary antecedents and neuropeptide intermediaries. Annals of the New York Academy of Sciences.

[CR49] Pearce E, Launay J, Dunbar RIM (2015). The ice-breaker effect: singing mediates fast social bonding. Royal Society Open Science.

[CR50] Pinquart M, Duberstein PR (2010). Association of social networks with cancer mortality: a meta-analysis. Critical Review of Oncology and Haematology.

[CR51] Rappoport R (1968). Pigs for the ancestors.

[CR52] Rodriguez-Laso A, Zunzunegui MV, Otero A (2007). The effect of social relationships on survival in elderly residents of a Southern European community: a cohort study. BMC Geriatrics.

[CR53] Saland LC, Chavez JB, Lee DC, Garcia RR, Caldwell KK (2008). Chronic ethanol exposure increases the association of hippocampal mu-opioid receptors with G-protein receptor kinase 2. Alcohol.

[CR54] Sarkar DK, Sengupta A, Zhang C, Boyadjieva N, Murugan S (2012). Opiate antagonist prevents μ- and δ-opiate receptor dimerization to facilitate ability of agonist to control ethanol-altered natural killer cell functions and mammary tumor growth. Journal of Biological Chemistry.

[CR55] Sayal K, Checkley S, Rees M, Jacobs C, Harris T, Papadopoulos A, Poon L (2002). Effects of social support during weekend leave on cortisol and depression ratings: a pilot study. Journal of Affective Disorders.

[CR56] Silk JB, Alberts SC, Altmann J (2003). Social bonds of female baboons enhance infant survival. Science.

[CR57] Silk JB, Beehner JC, Bergman TJ, Crockford C, Engh AL, Moscovice LR, Wittig RM, Seyfarth RM, Cheney DL (2009). The benefits of social capital: close social bonds among female baboons enhance offspring survival. Proceedings of the Royal Society of London.

[CR58] Silk JB, Beehner JC, Bergman TJ, Crockford C, Engh AL, Moscovice LR, Wittig RM, Seyfarth RM, Cheney DL (2010). Strong and consistent social bonds enhance the longevity of female baboons. Current Biology.

[CR59] Spence J (1954). One thousand families in Newcastle.

[CR60] Stiller J, Dunbar RIM (2007). Perspective-taking and memory capacity predict social network size. Social Networks.

[CR61] Strathern M (1971). The rope of Moka.

[CR62] Sutcliffe A, Dunbar RIM, Binder J, Arrow H (2012). Relationships and the social brain: integrating psychological and evolutionary perspectives. British Journal of Psychology.

[CR63] Tarr B, Launay J, Cohen E, Dunbar RIM (2015). Synchrony and exertion during dance independently raise pain threshold and encourage social bonding. Biology Letters.

[CR64] Tarr B, Launay J, Dunbar RIM (2016). Silent disco: strangers dancing in synchrony have an elevated pain threshold and feel socially close. Evolution and Human Behavior.

[CR65] Tilvis RS, Routasalo P, Karppinen H, Strandberg TE, Kautiainen H, Pitkala KH (2012). Social isolation, social activity and loneliness as survival indicators in old age: a nationwide survey with a 7-year follow-up. European Geriatric Medicine.

[CR66] Van Ree JM (1996). Endorphins and experimental addiction. Alcohol.

[CR67] Waxler-Morrison N, Hislop T, Mears B, Kan L (1991). Effects of social relationships on survival for women with breast cancer: a prospective study. Social Science & Medicine.

[CR68] Whitehead H (2000). Food rules: Hunting, sharing, and tabooing game in Papua New Guinea.

[CR69] Wiessner PW (2014). Embers of society: firelight talk among the Ju/’hoansi Bushmen. Proceedings of the National Academy of Sciences of the United States of America.

[CR70] Wittig RM, Crockford C, Lehmann J, Whitten PL, Seyfarth RM, Cheney DL (2008). Focused grooming networks and stress alleviation in wild female baboons. Hormones and Behavior.

[CR71] Young M (1971). Fighting with food.

[CR72] Zhang M, Kelley AE (2000). Enhanced intake of high-fat food following striatal mu-opioid stimulation: microinjection mapping and *fos* expression. Neuroscience.

